# Predicting Compression Pressure of Knitted Fabric Using a Modified Laplace’s Law

**DOI:** 10.3390/ma14164461

**Published:** 2021-08-09

**Authors:** Yetanawork Teyeme, Benny Malengier, Tamrat Tesfaye, Simona Vasile, Wolelaw Endalew, Lieva Van Langenhove

**Affiliations:** 1 Centre for Textile Science and Engineering, Department of Materials, Textiles and Chemical Engineering, Ghent University, 9052 Ghent, Belgium; Benny.Malengier@UGent.be (B.M.); Lieva.VanLangenhove@UGent.be (L.V.L.); 2 Ethiopian Institute of Textile and Fashion Technology, Bahir Dar University, Bahir Dar 6000, Ethiopia; tamrat_tsfy@yahoo.com (T.T.); enwolal@gmail.com (W.E.); 3 Fashion and Textiles Innovation Lab (FTILab+), HOGENT University of Applied Sciences and Arts, 9052 Ghent, Belgium; simona.vasile@hogent.be

**Keywords:** stretch fabric, interface pressure, Laplace’s law, compression garment

## Abstract

The aim of this study is to develop a mathematical model for the prediction of compression pressure based on fabric parameters, such as engineering stress, engineering strain and engineering modulus of elasticity. Four knitted compression fabrics with different fibrous compositions and knit structures were used. Rectangular-cut strips were employed for the force–elongation characterization of the fabrics. The experimental pressure values between the fabric and rigid cylinder were assessed using a Picopress pressure measuring device. The mechanical and physical parameters of the fabric that influence the interface pressure, such as strain, elasticity modulus/stress and thickness, were determined and integrated into Laplace’s law. A good correlation was observed between the experimental and calculated pressure values for all combinations of fabrics, mounted with variable tension on the cylinder. Over the considered range of pressures, the difference between the two datasets was generally less than 0.5 mmHg. The effect of washing after five, ten and fifteen washing cycles on the fabric–cylinder interface pressure was found to be significant.

## 1. Background

Graduated compression has been used in diverse medical applications for several years [1,2,3]. Compression garments are believed to assist in relieving ailments by providing evidence for their use in the sporting business [4,5]. However, the interface pressure applied by garments has not been accurately measured in the majority of research papers in this area [6,7]. Studies that have reported interface pressure measurements have commonly evaluated only the calf and the mid-thigh, which frequently reflect the exhibition of graduated compression [8,9,10].

Different postures and positions during the wear of compression garments induce variations in the garment–body interface pressure. The interface pressure exerted by garments of different sizes is an area that should still be investigated further. Studies have suggested that undersized garments exert more pressure than recommended size garments [11]. To quantify differences between the garments assessed, a complete pressure profile should be established. A lot has been done to quantify the pressure exerted by compression garments on lower body parts, however to the authors’ knowledge, there is no study that examines the pressure exerted by compression garments on the upper body of cyclists. This study aims to investigate the interface pressure between various sportswear compression fabrics and a rigid cylinder of size comparable to that of a human arm. In addition to this, it also looks at the relationship between the experimental values, measured by a Picopress instrument [12], and the predicted values obtained using the modified Laplace law that uses easy to measure fabric mechanical and physical parameters as input [13].

## 2. Materials and Methods

### 2.1. Materials

Four commercial knitted fabrics (Liebaert Marcel Nv, Deinze, Belgium) with 25–42% elastane, suitable for sports compression wear applications, were selected for this study. Table 1 lists the fiber composition, fabric structural parameters and means values (STDEV) of area density, thickness [14] and air permeability [15]. Area density of the fabrics varied from 150 g/m^2^ (fabric LB) to 310 g/m^2^ (fabric TL), they had a thickness between 0.29 mm (fabric LB) and 0.58 mm (fabric SW) and exhibited an air permeability with values between 74.93 mm/s (fabric DB) and 394.5 mm/s (fabric LB).

**Figure 1:**
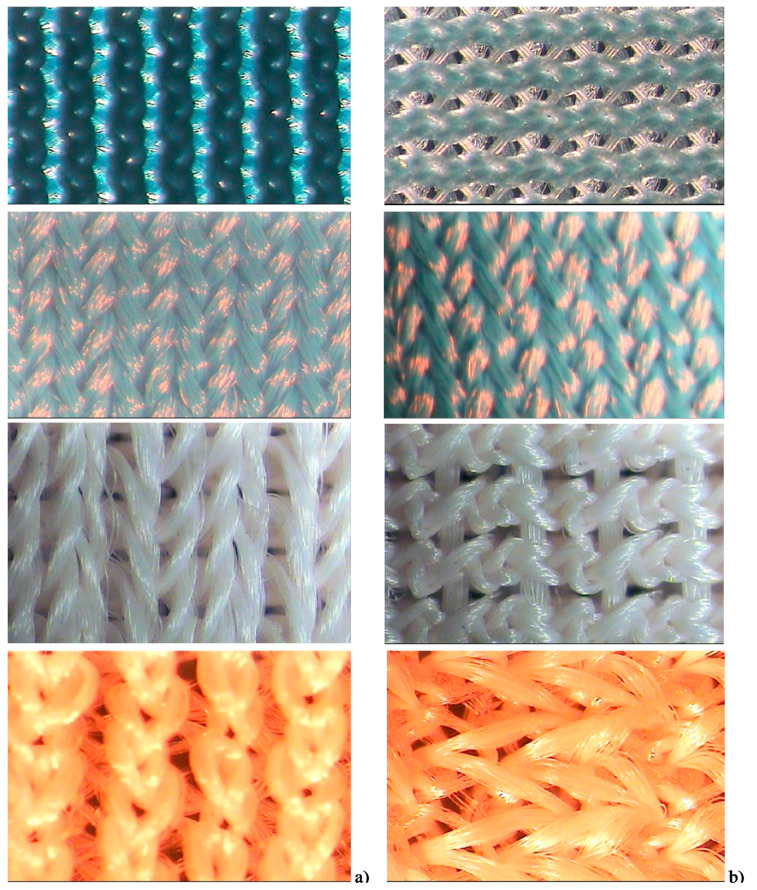
Structure of sample compression fabrics (LB, DB, TL and SW): technical face (**a**) and back face (**b**).

Figure 1 shows the technical face (a) and back face (b) surfaces of the compression fabrics (LB, DB, TL and SW). Usually, the fabric has raised wales on the face side of the fabric, whereas the inner side is smooth. A plain fabric back surface provides a comfortable smooth surface to the skin and also decreases stress concentration during compression. Moreover, the compression force was mostly developed by one-dimensional fabric stretching. This means that for producing effective pressure in compression garment design, the wale direction with Spandex or Lycra^®^ (Du Pont, Wilmington, DE, USA) is normally used [16,17].

### 2.2. Methods

Hereafter, all test methods are briefly described. First, a method is presented for the experimental assessment of the pressure (Section 2.2.1) exerted by the four fabrics on a rigid cylinder, approaching a human body part such as an arm. The test methods for the assessment of fabric modulus of elasticity (Section 2.2.3) [18] and theoretical prediction of interface pressure of the fabric–rigid cylinder are further presented (Section 2.2.4). Moreover, the effects of domestic washing after five, ten and fifteen wash cycles on the fabric–cylinder interface pressure were investigated (Section 2.2.2). Prior to testing, all samples were placed for at least 24 h in a conditioning chamber (Memmert, France), controlled at 21 ± 2 °C and a relative humidity of 65% ± 4% [19].

#### 2.2.1. Interface Pressure Evaluation

Adequate and stable pressure is crucial for compression garments. Extensibility and elastic recovery are important characteristics, due to which compression garments can exert continuous, constant pressure on the human body. The mechanical properties of the fabric and the curvature radius of the respective body parts are of great importance when constructing compression garments. An improper compression garment would influence the energy, work efficiency and health of the wearer. Insufficient pressure will limit efficacy, while too high a pressure will make people feel uncomfortable, cause breathing difficulty, and can also cause serious damage to health [20,21].

**Figure 2:**
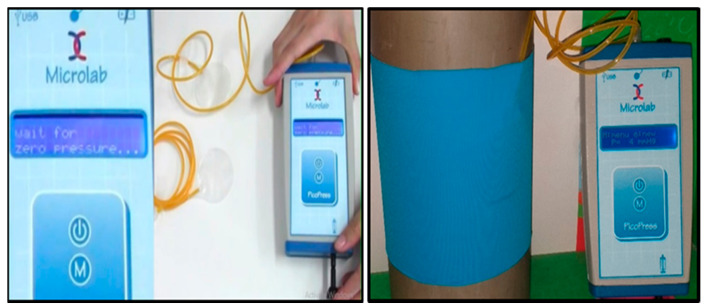
PicoPress^®^ pressure sensor device (**left**) used to measure fabric–rigid cylinder interface pressure in vitro (**right**).

Clothing pressure can be obtained by many methods, including theoretical calculations [22], simulations [23,24], direct tests, or indirect tests [25,26]. The garment pressure (mmHg) of the stretchable knitted fabrics was assessed using a PicoPress^®^ pressure sensor (Microlab Elettronica SAS, Padua, Italy) [12], which is shown in Figure 2. This instrument consists of a manometer connected to a probe, namely a flexible, circular plastic bladder with a diameter of 5 cm. This device is accurate, reliable and measures the actual pressure experienced by the wearer [27]. A rigid PVC (Polyvinyl chloride) cylinder of 11 cm diameter (34.56 cm circumference), approximating the circumference of an adult human upper arm, was used in this protocol. Fabric samples were cut and sewn into circular tubes, whose circumferences were 10%, 20% and 30% less than the circumference of the rigid PVC cylinder [28]. The fabric tube was mounted on the cylinder, the bladder (pressure sensor) was placed between the cylinder and the fabric (Figure 2) and the interface pressure of the cylinder–fabric was measured. For each sample, the static interface pressure was recorded at five different locations and the mean values (mmHg) were calculated.

#### 2.2.2. Effects of Washing on Pressure

The effect of domestic washing after five, ten and fifteen wash cycles on the fabric–cylinder interface pressure was investigated [29]. The pressure exerted by the circular sample on the rigid cylinder was also tested after 5, 10 and 15 domestic washes of the fabric tube, performed according to ISO 6330:2012(E) [29]. After each washing cycle (of a duration of 30 min), the fabrics were flat-dried at chamber temperature and the interface pressure of the dry fabric tubes was subsequently measured.

#### 2.2.3. Fabric Elasticity and Flexural Modulus

**Figure 3:**
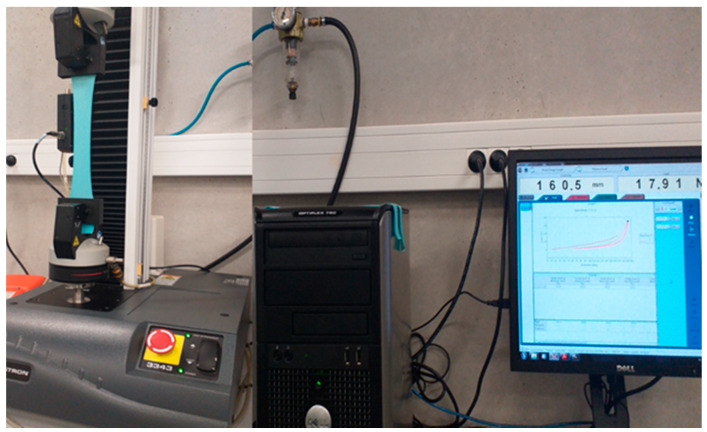
Strip tests for stretch-recovery measurement using an Instron 3334 Tensiometer.

Measurements of fabric elasticity and the flexural modulus of elasticity were carried out using an Instron 3334 Tensiometer (Instron, Boechout, Belgium) (Figure 3). Assessment of the stretch recovery was determined according to EN 14704-1:2005 [18]. The fabric strip of 50 mm width was tested under a gauge length of 100 mm and at a constant test speed of 500 mm/min. The load (N) was preset at a value determined as a function of the fabric’s elastane content and the value of strain (%) was determined for five cycles. The strain value of the first cycle was used in further calculations. For each fabric, five specimens were tested, and the mean values were calculated [30].

#### 2.2.4. Theoretical Method for Prediction of Compression

To calculate the pressure applied by the compression garments, a method for validating the reliability of Laplace’s law was investigated (Section 3.4.1). Furthermore, the physical and mechanical properties (such as fabric elasticity, weight and thickness) of nylon/elastane knitted fabrics were studied and compared to confirm their capability to prove satisfactory compression. Such a study is necessary to understand whether a fabric is suitable for developing a compression garment for a determined application and for estimating the required compression force needed when designing a custom-made compression garment with sports compression fabrics.

## 3. Results and Discussion

### 3.1. Interface Pressure Evaluation

The results showed that the tricot with pillar stitch (TL) exerted the highest pressure on the cylinder, with average values between 5.7 mmHg (at 10% stretch) and 16.6 mmHg (at 30% stretch). The maximum attained pressure in this study falls under the moderator compression category descriptor and can help to reduce Deep Vein Thrombosis (DVT) in normal-risk patients, especially during periods of long travel. Previous studies also showed that patients with symptoms of mild venous insufficiency benefit from wearing compression garments providing a pressure of 10–20 mmHg [31]. However, to the authors’ knowledge, there is no known study available which quantifies the pressure produced by compression garments and the interactions between the garment and the upper body parts of a cyclist. The maximum pressure attained in this study could reduce the range of motion during cycling, improve body stability, support the muscles of cyclists and improve the blood flow. The extent of pressure depends on the garment fit and construction, the size and shape of the part of the body to which it is applied, structure and physical properties of the fabric and the type of sport activity. The future study will entail an interaction of size and shape of body parts of cyclists on pressure applied and the use of this compression garment for treating different venous diseases.

### 3.2. Effects of Washing on Pressure

**Figure 4:**
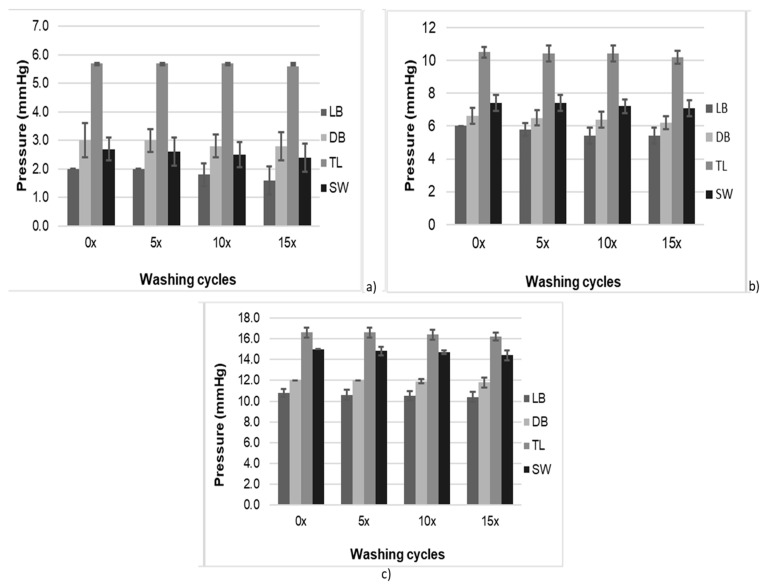
Pressure exerted by the 10% stretched fabrics (**a**), 20% stretched fabrics (**b**) and 30% stretched fabrics (**c**) on the tube after 5, 10 and 15 washing cycles.

Figure 4 shows the pressure in mmHg exerted by the knitted fabrics on the cylinder at zero washing cycles (noted as 0×). Among the tested fabrics, the tricot with pillar stitch (TL) mounted on the cylinder at 30% stretch exhibited the highest pressure (c) before washing (0×). This fabric had a greater compact structure and higher areal density, and consequently led to more pressure than other knit structures. The influence of various numbers of washing cycles (5×, 10×, 15×) on the pressure exerted by fabrics mounted at (a) 10%, (b) 20% and (c) 30% on the cylinder can be also seen in Figure 4.

Table 2 shows the pressure loss (in %) after 5, 10 and 15 washing cycles. For instance, at 30% stretch and after 15 washes, the pressure of all four knitted structures was reduced by 0.4, 0.2, 0.4 and 0.2 mmHg. After 5 washes, a pressure loss of 0.2, 0.0, 0.0 and 0.2 mmHg was noticed for the tricot single face LB, tricot double face DF, tricot with pillar stitch TL and tricot 1 × 1 SW, respectively. In general, tricot DB and tricot TL retained better pressure after 5, 10 and 15 washes than tricot LB and tricot SW, but not tricot 1 × 1 SW, which had a better pressure retained than DF at 20% stretch. In general, there was not much difference in terms of pressure loss between the four knitted structures after 5 washes, but this difference was quite significant after 10 and 15 washes, which suggests that the knitted stretchable fabrics tend to lose their compression ability after multiple washes. Moreover, a *t*-test (α = 0.05) showed significant differences between the pressure exerted by the fabric on the cylinder due to levels of stretch (*p* < 0.05).

### 3.3. Fabric Elasticity

**Figure 5:**
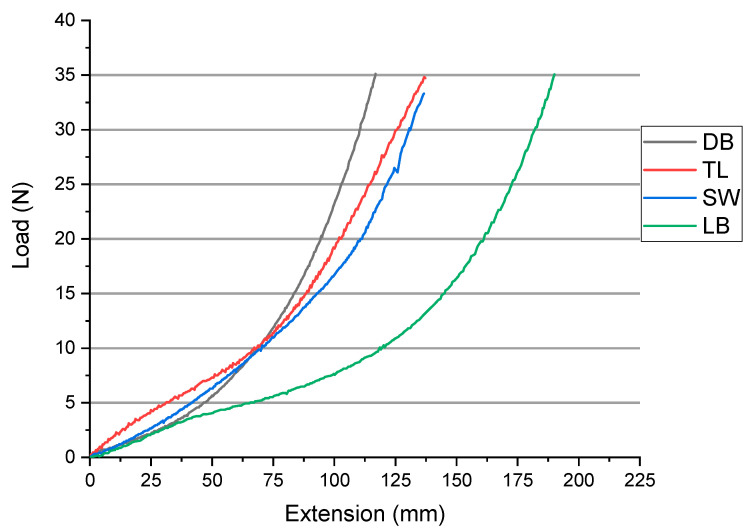
Load-extension curves for the fabrics DB, TL, SW and LB.

Double knits (DB) are in general more stable and heavier than single knits due to the opposite loop orientation of the double guide bar patterns. Single knit structures are dimensionally unstable and split easily when damaged. It is also quite evident in the strain of fabrics (Figure 5) that the tricot single face (LB) has the highest strain (190 mm) at maximum load of cycle 1, due to its looser structure (Figure 1) that makes it stretch quite easily. Similarly, tricot double face (DB) with the most compact structure exhibited the lowest strain (116 mm).

### 3.4. Theoretical Method for Prediction of Compression

#### 3.4.1. Interface Pressure Model

**Figure 6:**
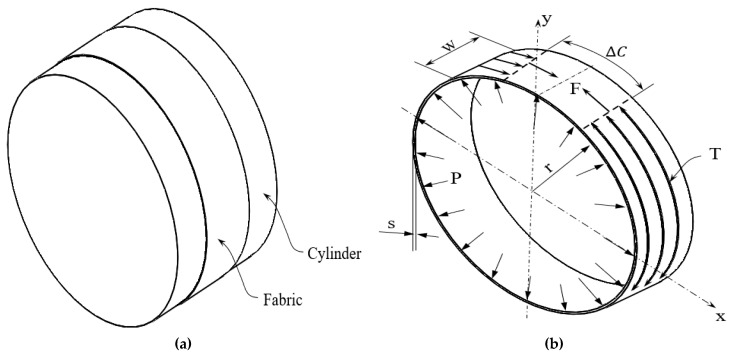
(**a**) PVC cylinder and fabric interface. (**b**) Forces present: pressure (P) and tension (T) for a cylinder with radius (r).

The predictive pressure values of the stretch fabrics were calculated by applying Laplace’s law [32,33]. In the investigation, the geometric form of the PVC cylinder (Figure 6) was assumed to represent the human body figure (arm).

**Figure 7:**
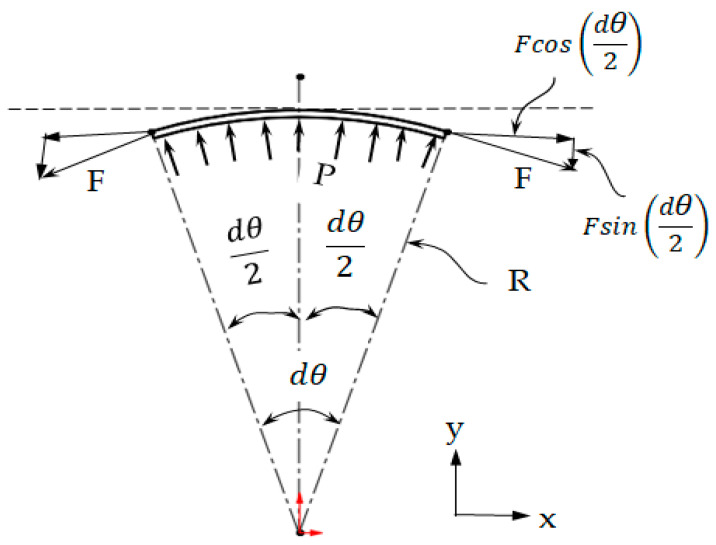
Differential circumference of fabric on the cylinder.

Laplace’s law follows from taking the differential circumference, as shown in Figure 7. The derivation is as follows. From the principle of static equilibrium, the pressure applied to a differential area can be given as $PdA-2Fsin\frac{d\theta }{2}=0$, with *dA* being the area on which the pressure works. For small angles, we have $sin\frac{d\theta }{2}\approx \frac{d\theta }{2}$, so we can rearrange this as PdA=Fdθ.

Integrating this equation over all angles, constant pressure was assumed:

(1) $$w2\pi rP=\int_{0}^{2\pi }PdA=\int_{0}^{2\pi }Fd\theta =F2\pi$$

This can be rewritten by assuming that the force is the tension force times the width of the sample, as indicated in Equation (2):

(2) $$P=\frac{F2\pi }{w2\pi r}=\frac{Tw2\pi }{w2\pi r}=\frac{2\pi T}{C}=\frac{T}{r}$$

where *P* is the surface pressure on the PVC cylinder (Pa), *F* is the internal force of the fabric (N), *T* the fabric tension applied on the cylindrical surface (N/m), *C* is the circumference of the fabric (m), *w* is the fabric width (m), and *r* is the curvature radius of the cylindrical surface (m). This equation is known as Laplace’s law [11,34].

Stress developed on the fabric due to the surface pressure can be determined as the ratio of applied force to the fabric cross-sectional area, $A_{s}$ (m^2^) (Equation (3)), which can be given as:

(3) $$\sigma =\frac{F}{A_{s}}=\frac{Tw}{sw}=\frac{T}{s}$$

where σ is engineering stress (Pa) of the test fabric, and *w* and *s* are fabric width and fabric thickness (in m), respectively.

Tension can hence be found as a function of the resisting stress and thickness of the fabric, as T=σs. Substituting it into Equation (2) gives:

(4) $$P=\frac{\sigma s2\pi }{C}=\frac{E\epsilon s2\pi }{C}$$

Here, we assumed that stress and strain obey Hooke’s law (Equation (5)) in the elastic region of the fabric:

(5) σ=Eϵ

where *E* is engineering Young’s Modulus (Pa) and ϵ is the engineering strain (m/m).

The strain for the cylindrical model can also be determined from the change in circumference, as shown in Equation (6):

(6) $$\varepsilon =\frac{C_{f}-C_{i}}{Ci}$$

where $C_{f}$ is the initial test fabric circumferences and the final test fabric measured before and after stretching.

Prediction model of the fabric stress:

The test data obtained from Instron based on EN 14704-1 [17] were related using a third-order polynomial equation (Equation (7)), which provides the best fit:

(7) $$\sigma \left(\varepsilon \right)=a_{1}\varepsilon^{3}+a_{2}\varepsilon^{2}+a_{3}\varepsilon +b$$

where $a_{1},a_{2},a_{3}$ coefficients and b is a constant number.

Substituting Equation (6) into Equation (7), it gives:

(8) $$\sigma \left(\varepsilon \right)=a_{1}{\left(\frac{C_{f}-C_{i}}{Ci}\right)}^{3}+a_{2}{\left(\frac{C_{f}-C_{i}}{Ci}\right)}^{2}+a_{3}\left(\frac{C_{f}-C_{i}}{Ci}\right)+b$$

The resulting pressure, P (Pa), was calculated using Equations (4) and (8) for stress and compared with the measured pressure:

(9) $$P=\frac{s2\pi }{C}\left(a_{1}{\left(\frac{C_{f}-C_{i}}{C_{i}}\right)}^{3}+a_{2}{\left(\frac{C_{f}-C_{i}}{C_{i}}\right)}^{2}+a_{3}\left(\frac{C_{f}-C_{i}}{C_{i}}\right)+b\right)$$

#### 3.4.2. Influence of Fabric Mechanical Properties on Interface Pressure

**Figure 8:**
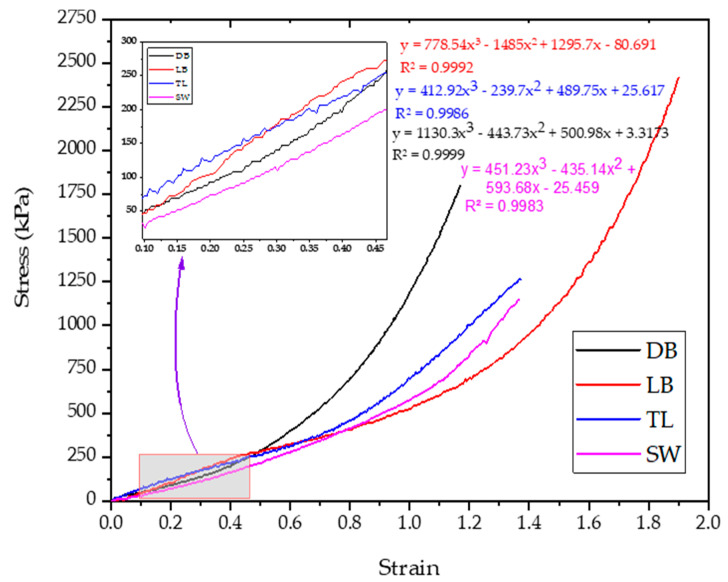
Stress–strain curves for the tested samples.

The stress–strain curves were analyzed, and Figure 8 presents the obtained tension for a specific strain found throughout the stress–strain curves. As presented in the experimental procedure, the knitted fabric tube extends when worn (Figure 2). The applied tension at these extension values was obtained from the stress–strain curve.

### 3.5. Measurements and Comparison of the Pressure with Prediction Values

In this study, we applied the mathematical model (Equation (9)) and compared it with the experimental pressure (PE). The measured (PE) and predicted values (P) of each fabric with various extension levels (i.e., 1 = 10%, 2 = 20% and 3 = 30%) are shown in Table 3. The pressure results using the developed prediction model derived as (Equation (9)) were statistically analyzed and compared with the experimental pressure measurement results.

**Figure 9:**
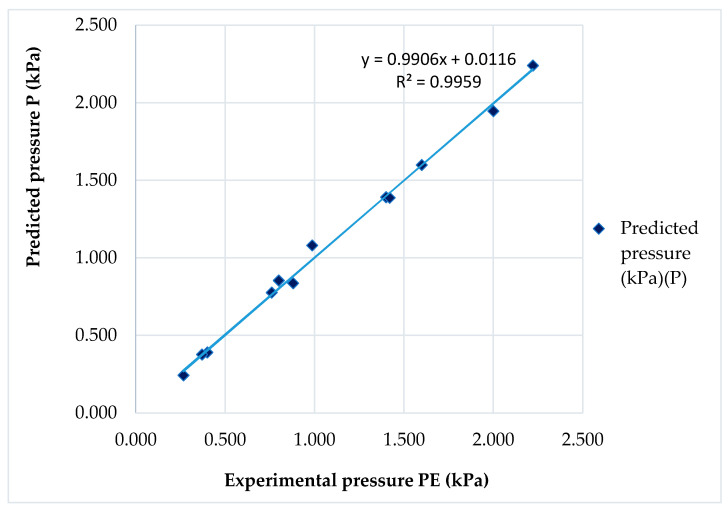
Comparison of predicted compression pressure (P) with experimental results (P_E_).

Linear regression analysis was used to investigate how much the independent variable (experimental pressure) explained the dependent variable. Figure 9 shows the coefficient of determination value (R-squared value) of the prediction model (P), explaining 99.5% of the experimental results. In addition, the accuracy of a developed prediction model (P) against experimental pressure (PE) was assessed. The errors between predicted and experimental values ranged between 0.06% and 9.42%. The absolute values of their relative errors were very small, except for SW2 (9.42%), LB1 (−9.02%) and LB2 (6.74%). This can be caused by an extension (%), the radius of the area on which pressure is applied and other mechanical parameters of the fabric, such as friction properties on the PVC tube [35,36]. However, according to our analysis, this value is acceptable for the development process of interface compression pressure.

### 3.6. The Influence of Extension and Young’s Modulus on Interface Pressure

**Figure 10:**
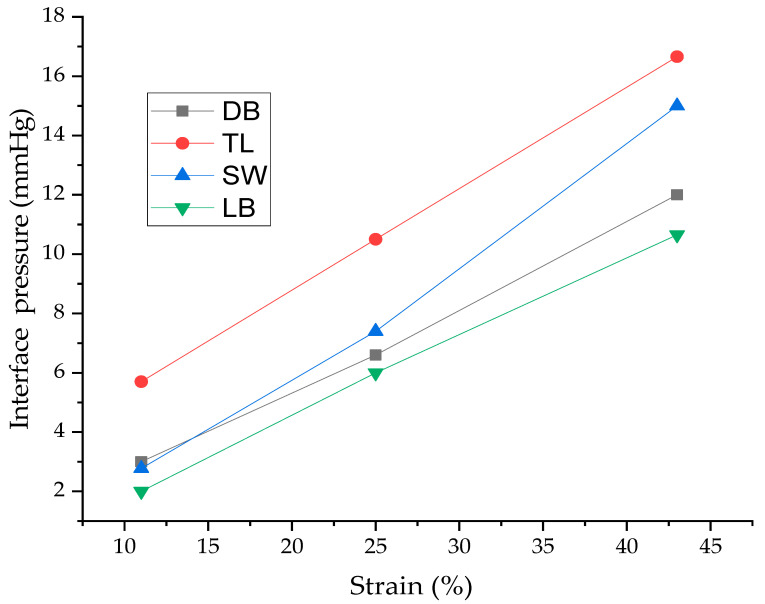
Interface pressure applied by the different samples at determined strain values of 10%, 20% and 30%.

As illustrated in Figure 10, at the same strain value, the interface pressure increases when Young’s modulus increases (Table 3). This variation is related to the mechanical behavior of the sample fabrics. The greater the Young’s modulus (as in the case of knitted fabric TL), the higher the tension needed to obtain the desired strain. Consequently, the interface pressure becomes higher. Furthermore, these results clearly follow Laplace’s law, where pressure applied by a fabric with a greater elastic modulus is higher than that exerted by a fabric with a lower elastic modulus for a similar strain.

With respect to extension, Figure 10 shows that at the same strain value, the interface pressure values increase when Young’s modulus increases. This difference can be related to the behavior of the knitted samples.

### 3.7. Influence of Fabric Thickness on Interface Pressure

The mechanical properties of textile materials are influenced by fabric thickness, as presented in Table 1. Figure 10 also shows the effect of fabric thickness on the interface pressure applied. Except for fabric SW, it is clear that the thicker the compression fabric (i.e., fabric TL and DB), the greater the interface pressure. This can be explained by the fact that at the same fabric circumference, if the other parameters are kept constant, the thickest knitted fabric generates more internal force to the rigid cylinder.

## 4. Conclusions

Compression clothing is broadly utilized nowadays in several medical and sports applications. However, there is a lack of studies on the interface pressure applied by the different sized garments at different extension levels. The scope of this investigation was to assess and predict the interface pressure generated by a knitted fabric on a rigid tube, based on a pressure measurement device and a revised Laplace’s law. In order to predict the interface pressure generated by a knitted elastic garment on a human body part, the basic Laplace’s law is inadequate, as the tension remains unknown. Therefore, it was necessary to present adjustments to this law to recast it in terms of extension. For predicting the interface pressure, the fabric–cylinder circumference, knitted fabric thickness, applied for extension, and its corresponding strain were included. The theoretical results obtained with this modified Laplace’s law model were presented and compared to that of the experimental pressure value. We found that the prediction of the model was sufficiently good for use in compression garment design. Moreover, to verify the validity of the proposed model, experimental and predicted data were compared and very low error values were found.

As limitations, we need to mention that this study included a small number of fabric types and cylinder circumferences. A further analysis should include a higher number of fabrics and more circumference. The interface pressure was measured using a rigid cylinder approach, which is frequently used in the literature, but the human body is not rigid and has anatomical variation. Due to the non-uniformity of body shapes, the pressure exerted by a garment with a given tension is not uniform and is distributed differently over the various areas of the body as well. Nevertheless, the presented results aid in the selection of materials for good-fitting compression garments.

## Figures and Tables

**Table 1:** Fabric information and properties.

Sample Code	Nylon/Elastane Composition (%)	Fabric Construction	Area Density (g/m^2^)	Air Permeability (mm/s)	Thickness (mm)	Wales/cm	Courses/cm
LB	74/26	Warp knit— Tricot single face	150	394.5 (11.59)	0.29 (0.008)	31	37
DB	58/42	Warp knit— Tricot double face	200	74.93 (6.79)	0.39 (0.00)	29	39
TL	65/35	Warp knit—Tricot with pillar stitch	310	183.25 (6.94)	0.55 (0.01)	22	26
SW	75/25	Warp knit— Tricot (1 × 1)	247	314.0 (27.18)	0.58 (0.01)	22	38

**Table 2:** Pressure loss after 5, 10 and 15 washing cycles of the fabrics LB, DB, TL and SW mounted at 10%, 20% and 30% stretch on the rigid cylinder.

Fabric Name	Pressure Losing Percentage (%)								
	Washes 5×			Washes 10×			Washes 15×		
	10% Stretch	20% Stretch	30% Stretch	10% Stretch	20% Stretch	30% Stretch	10% Stretch	20% Stretch	30% Stretch
Tricot single face (LB)	0.00	3.33	1.85	10.00	10.00	2.78	20.00	10.00	3.70
Tricot double face (DB)	0.00	1.52	0.00	6.67	3.03	0.83	6.67	6.06	1.67
Tricot with pillar stitch (TL)	0.00	0.95	0.00	0.00	0.95	1.20	1.75	2.86	2.41
Tricot (1 × 1) (SW)	3.60	0.00	1.33	7.21	2.70	2.00	10.81	4.05	4.00

**Table 3:** Measurement of engineering strain, stress, modulus and predicted compression.

Fabric Code	Measured Pressure P_E_^(1)^ (mmHg)	Engineering Strain ε	Engineering Stress δ (kPa)	Young’s Modulus E (kPa)	Experimental Pressure P_E_^(2)^ (kPa)	Predicted Pressure P (kPa)	% Error [(|P − P_E_|) /P_E_] × 100
DB _1_	3.0	0.111	55.054	495.49	0.40	0.39	2.50
DB _2_	6.6	0.249	118.047	473.90	0.88	0.84	4.89
DB _3_	12.0	0.429	225.496	526.16	1.60	1.60	0.06
TL _1_	5.7	0.111	77.638	698.74	0.76	0.78	2.11
TL _2_	10.5	0.249	139.112	558.46	1.40	1.39	0.64
TL _3_	16.6	0.429	223.967	522.59	2.22	2.24	0.86
SW _1_	2.7	0.111	35.754	321.79	0.37	0.38	1.89
SW _2_	7.4	0.249	102.405	411.11	0.99	1.08	9.42
SW _3_	15.0	0.429	184.584	430.70	2.00	1.95	2.70
LB _1_	2.0	0.111	46.010	414.09	0.27	0.24	9.02
LB _2_	6.0	0.249	161.954	650.16	0.80	0.85	6.74
LB _3_	10.8	0.429	263.138	1.420	1.39	1.39	2.29

Indices 1/2/3 represent extension levels of 10%/20%/30% of the fabrics DB, TL, SW and LB. ^(1)^ Values correspond to P_E_ (mmHg), as indicated in Figure 4, for 0x washing cycles. ^(2)^ P_E_ values (mmHg) in the first column converted to kPa.

## Data Availability

Data sharing is not applicable to this article.
